# Probing the Intrinsic Strain in Suspended Graphene Films Using Electron and Optical Microscopy

**DOI:** 10.1002/advs.202305366

**Published:** 2023-12-06

**Authors:** Kishan Thodkar, Milivoj Plodinec, Fabian Gramm, Karsten Kunze

**Affiliations:** ^1^ Micro‐ & Nanosystems Department of Mechanical & Process Engineering ETH Zurich Tannenstrasse 3 Zurich 8092 Switzerland; ^2^ ScopeM ETH Zurich Otto‐Stern‐Weg 3 Zurich 8093 Switzerland

**Keywords:** chemical vapor deposition (CVD), electron backscatter diffraction (EBSD), Raman spectroscopy, suspended graphene, transmission electron microscopy (TEM)

## Abstract

Quantifying the intrinsic properties of 2D materials is of paramount importance for advancing their applications. Large‐scale production of 2D materials merits the need for approaches that provide direct information about the role of growth substrate on 2D material properties. Transferring the 2D material from its growth substrates can modify the intrinsic properties of the asgrown 2D material. In this study, suspended chemical vapor deposition (CVD) graphene films are prepared directly on their growth substrates in a high‐density grid array. The approach facilitates the quantification of intrinsic strain and doping in suspended CVD graphene films. To achieve this, transmission electron microscopy and large‐area Raman mapping are employed. Remarkably, the analysis reveals consistent patterns of compressive strain (≈−0.2%) both in the diffraction patterns and Raman maps obtained from these suspended graphene films. By conducting investigations directly on the growth substrates, the potential influences introduced during the transfer process are circumvented effectively. Consequently, the methodology offers a robust and reliable means of studying the intrinsic properties of 2D materials in their authentic form, uninfluenced by the transfer‐induced alterations that may skew the interpretation of their properties.

## Graphical abstract

Characterizing single layer graphene (SLG) films directly on their growth substrates is highly beneficial to study their intrinsic properties. Charge doping and strain are among the prominent properties of interest to assess SLG quality. In this work, the use of selected area electron diffraction and Raman spectroscopy as means to quantify intrinsic strain in chemical vapor deposition grown SLG films is demonstrated.

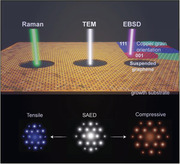

## 1 Introduction

Large‐area single and multi‐layer 2D materials can be synthesized using chemical vapor deposition (CVD) and epitaxial methods on various growth substrates.^[^ ^1^, ^2^ ^]^ Quantifying the quality of the 2D material films using various techniques such as Raman spectroscopy, transmission and scanning electron microscopy (TEM, SEM), electrical transport, and terahertz spectroscopy has provided a significant understanding of their properties.^[^ ^3^, ^4^, ^5^, ^6^, ^7^ ^]^ Considering the single‐ and few‐atom thick nature of these 2D materials, their intrinsic properties are highly susceptible to adsorbents and process conditions.^[^ ^8^, ^9^ ^]^ Polymeric adsorbents (e.g., photoresist) can be introduced during lithography and introduce charge doping in graphene films. Moreover, process conditions such as thermal annealing have been reported to introduce strain in CVD graphene films.^[^ ^10^, ^11^ ^]^ Encapsulation and suspension of graphene films and other 2D materials have resulted in low charge doping and intrinsic strain.^[^ ^12^, ^13^, ^14^ ^]^ The encapsulation methods focus on the heterostructure formation of graphene and hexagonal Boron Nitride (hBN), resulting in low surface roughness, residual charge doping, and strain.^[^ ^12^, ^15^ ^]^ The suspension methods focus on supporting the graphene film at the periphery (contact region).^[^ ^13^, ^14^ ^]^ Both these methods target isolating 2D materials from the oxide surface to minimize charge trap‐induced scattering and have been primarily used to study the 2D materials after transferring them from their growth substrate (e.g., copper, nickel, platinum, or others) to device substrate (e.g., Si/SiO_2_ substrates or TEM grids).

Any transfer method risks influencing the intrinsic properties of as‐synthesized CVD‐grown 2D materials and losing information about 2D material properties such as intrinsic strain, defects, crystallinity, and orientation. In addition, after the 2D material transfer, it is no longer possible to characterize the direct role of the growth substrate on CVD graphene film quality. Separating the CVD graphene films from their growth substrates further disconnects potentially relevant local information on the growth substrate like crystallinity, topographical features, grain boundaries, and defects.

With these in consideration, it is crucial to distinguish the “as‐synthesized” and “transferred” 2D material as two separate entities. Distinguishing them is beneficial in characterizing and optimizing 2D material properties. One approach to studying the 2D material properties in their intrinsic state is to perform the characterization directly on the growth substrates. However, non‐covalent interactions with the underlying growth substrates can influence intrinsic properties and limit signal acquisition.^[^ ^16^ ^]^ To overcome these limitations, suspending the 2D materials directly on their growth substrates provides a simple solution. We developed a simplified process to suspend single‐layer CVD graphene‐on‐grid in a high‐density grid array to study the intrinsic properties of CVD graphene films. Zheng et al.^[^ ^17^ ^]^ have reported a similar approach on the application of suspended graphene films as thin membrane support for high‐resolution electron microscopy. The suspended graphene‐on‐grid makes them accessible and compatible for characterization using a wide range of commonly used optical and electron microscopy methods. This method allows for studying the intrinsic material and growth substrate properties such as intrinsic strain, doping, defect density, and the influence of growth substrate crystallinity on graphene grain orientation, thereby circumventing the need for material transfer. Our method uses the established UV photolithography (PL) protocol and the commonly used copper etching method.^[^ ^18^, ^19^ ^]^ By combining TEM and large‐area Raman mapping, we were able to probe the intrinsic strain and doping in suspended CVD graphene films. We quantified the selected area electron diffraction peak features and found the presence of compressive strain in graphene. By performing large‐area Raman mapping, we validated the presence of compressive strain and quantified defects resulting from imaging of graphene films using electron microscopy.

## 2 Results

**Figure 1:**
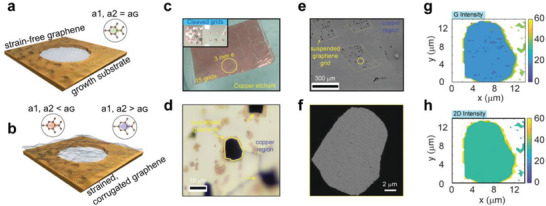
High‐density suspended, single‐layer CVDG grid array overview. Schematic of a) flat, strain‐free suspended CVDG film, and b) strained, corrugated, suspended CVDG film on growth substrate. Optical images of c) high‐density, suspended CVDG film grids floating on copper etchant solution. Inset: Image of six prepared grids taken after etching and drying process. d) Optical image of a suspended CVDG grid region R5. e) SEM image of a part of the CVDG grid array. The preselected, individual grid area R5 is highlighted using a yellow circle. f) A TEM image of region R5. The brighter region in the TEM image is an indication of fully etched windows in the copper growth substrate. Raman maps of g) G, and h) 2D peak intensity of the graphene‐on‐grid in region R5. The color bar indicates the peak intensity count.

**Figure 2:**
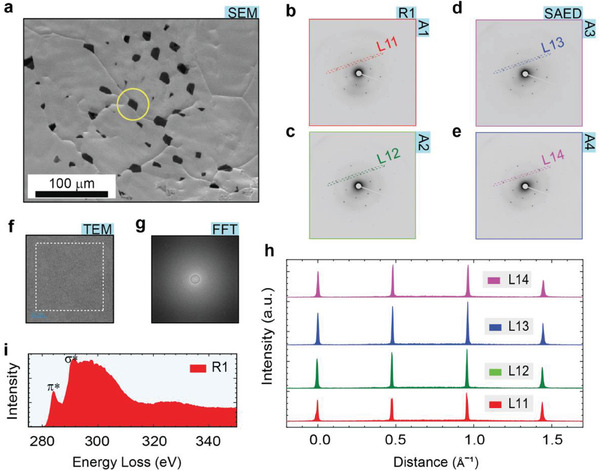
Characterization of growth substrate and graphene crystallinity within a single suspended graphene‐on‐grid region. a) SEM image of the CVDG grid region. CVDG grid region R1 is highlighted by yellow circle. b–e) SAED patterns were acquired within region R1 at areas A1–A4 with line profile regions L11–L14 highlighted in dotted lines. f) TEM image of region R1, and g) the FFT of the region highlighted using white dotted square. Note: the sixfold symmetry visible in the FFT image. h) Intensity profiles of diffraction spots along lines L11–L14 highlighted in SAED patterns of R1 region, and i) EEL spectrum of C K edge.

In **Figure** **1**a, the schematic illustration of a strain‐free (Figure 1a) and strained (Figure 1b) suspended CVD graphene film on the copper growth substrate is depicted. Note the changes in the graphene unit cell length scales under strain‐free (green), compressive (red), or tensile (blue) strain configurations. CVD graphene is synthesized on commercially available polycrystalline copper foils (≈25 µm thickness); see the experimental section for additional details related to CVD synthesis and the fabrication process of suspended graphene‐on‐grid. An optical image of the etching process is presented in Figure 1c. An overview of the grid fabrication process is presented in Figure S1a (Supporting Information). In the inset of Figure 1c, six successfully cleaved CVDG/Cu grids (*⌀*≈3 mm) are presented. An optical image of an individual, suspended graphene‐on‐grid region (area ≈8 × 10 µm^2^) is presented in Figure 1d. The unique periphery of the grid regions is beneficial in identifying the regions during SEM (Figure 1e), TEM (Figure 1f), and Raman characterization. The suspended architecture wherein the graphene film is supported only at the periphery is beneficial for Raman characterization of the suspended graphene without the major influence of the underlying copper growth substrate. The large‐area Raman mapping of suspended CVD graphene‐on‐grid region can be performed, and the respective G (Figure 1g) and 2D (Figure 1h) band intensity is presented. The 2D intensity profiles of graphene characteristics Raman bands provide insight into the uniformity of the peak intensity within the suspended graphene region. An increase in the intensity of 2D Raman band (*I*_2D,_ Figure 1h) compared to G band intensity (*I*_G,_ Figure 1g), i.e., the *I*_2D_/*I*_G_ ratio ≈two is observable. Representative Raman spectra collected in region R5 is presented in Figure S2 (Supporting Information).

### 2.1 Native Growth Substrate and Graphene Crystallinity of Graphene‐on‐Grid Regions

After the graphene‐on‐grid fabrication, suspended graphene‐on‐grid regions were identified and characterized using large‐area Raman mapping. The optical images of randomly selected graphene‐on‐grid regions for this study are presented in Figure S3 (Supporting Information). The graphene‐on‐grid regions were characterized using TEM and the copper growth substrate was characterized using electron backscatter diffraction. After TEM and EBSD mapping, Raman mapping of the graphene‐on‐grid regions R1–R7 was repeated.

This approach allows for the pre‐characterization of suspended graphene films before and after their electron beam irradiation. The TEM techniques allow for characterizing the crystallinity of the suspended graphene‐on‐grid regions. In **Figure** **2**a, an EBSD crystal orientation map (color‐coded for substrate normal direction and grey levels for Confidence Index combined) and the SEM image (inset) of the copper growth substrate are presented. Note the various crystallographic orientations of the underlying copper growth substrate. Additional EBSD orientation maps of the growth substrate are presented in Figure S4 (Supporting Information). A suspended graphene‐on‐grid region R1 identified during optical imaging is noticeably located at the boundary of 112 (pink) and 101 (green) grain (yellow circle). The dark regions within Figure 2a denote holes in the etched copper grid with the graphene film freely suspended. Multiple selected area electron diffraction (SAED) patterns were collected to probe the crystallinity and uniformity of the graphene region. The SAED patterns acquired within the suspended graphene‐on‐grid region R1 are presented in Figure 2b–e. Line profiles L11–L14, annotated as red dotted lines, depict the rotation of the diffraction pattern with respect to the horizontal axis. The difference in the rotation angle between L11–L14 was estimated to be < 1°. The six‐fold symmetry observable in all diffraction patterns is indicative of single layer and single crystalline nature of the suspended graphene film.^[^ ^5^, ^17^ ^]^ A TEM image and the fast Fourier transform (FFT) of the TEM image of region R1 are presented in Figure 2f,g. Note the six‐fold symmetry is also visible in the FFT image (see. Figure 2g). In the intensity profiles L11–L14 (Figure 2h), we can notice the overlap in the stacked diffraction spots acquired in multiple regions within the suspended graphene‐on‐grid region R1. The electron energy core‐loss spectrum of C‐k edge (EELS) of region R1 (Figure 2i) additionally confirms the presence of graphene.

**Figure 3:**
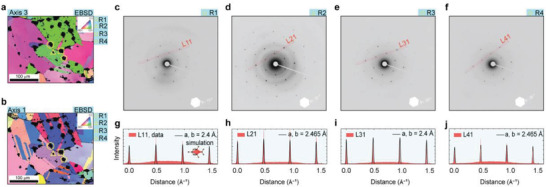
Characterization of growth substrate and graphene crystallinity across multiple suspended graphene‐on‐grid regions. a,b) EBSD orientation maps of the CVDG grid area color‐coded with respect to foil normal (axis 3, a), and in‐plane (axis 1, b) with the selected graphene‐on‐grid regions highlighted as yellow circle (R1), triangle (R2), square (R3), and pentagon (R4). c–f) SAED images of the CVDG regions R1–R4. Inset: The white hexagon indicates the rotation angle of SAED pattern with respect to the horizontal axis. g–j) Intensity profile of diffraction spots along the lines L11–L41 as indicated in c–f) of regions R1–R4 (in red). Representative intensity profile extracted from simulation is overlaid on top of the intensity profile with black line.

Additional SAED patterns from the surrounding graphene‐on‐grid regions R1–R7 were collected for comparison (see Figures S5–S10, Supporting Information). In **Figure** **3**, the EBSD orientation maps of the copper substrate are color‐coded with respect to crystal directions aligned along the foil normal direction (axis 3, Figure 3a) and along an in‐plane direction (axis 1, Figure 3b).

The orientation maps for different reference directions complement each other in identifying separate grains (and twins), which are all colored green in Figure 3a because they all have the [110] direction nearly normal to the foil surface. The graphene‐on‐grid regions R2 (triangle), R3 (square), and R4 (pentagon) are highlighted in Figure 3a,b. The SAED patterns collected in regions R1–R4 are presented in Figure 3c–f. Furthermore, additional SAED patterns within the graphene‐on‐grid regions R1–R7 are presented in Figures S5–S10 (Supporting Information). Inset: The rotation angle of the SAED patterns with respect to horizontal axis are highlighted using the white hexagon. The SAED rotation angles of R1 ≈19°, R2 ≈32°, R3 ≈20°, and R4 ≈36° were estimated (with respect to an arbitrarily chosen horizontal reference line). Note that within the region R1, the SAED rotation angle is within ± 1° (see Figure S11, Supporting Information). The influence of the copper crystallinity can account for such changes in SAED rotation angle across different grid regions (see Figures S12 and S13, Supporting Information).^[^ ^20^, ^21^ ^]^

The intensity profiles of diffraction spots along L11–L41 obtained from the graphene‐on‐grid regions R1–R4 are presented in Figure 3g–j. Simulated intensity profiles (black line, considering equivalent unit cell lengths “a” and “b”) were overlaid on the intensity profiles of diffraction spots obtained experimentally from SAED patterns in regions R1–R4 to approximate the equivalent unit cell length of the graphene. Additional details related to the simulated intensity profiles extraction are presented in Experimental Section. We can notice that for regions R1 and R3, the simulated intensity profiles match with unit cell length a, b = 2.4 Å and a, b = 2.465 Å for region R2 and R4, respectively. These observations are indicative of intrinsic compressive strain in the graphene‐on‐grid regions. We simulated the intensity profiles to quantify the influence of compressive and tensile strain in the suspended graphene‐on‐grid regions.

### 2.2 Intrinsic Compressive Strain Analysis Using Electron Diffraction Patterns Across Graphene‐on‐Grid Regions

**Figure 4:**
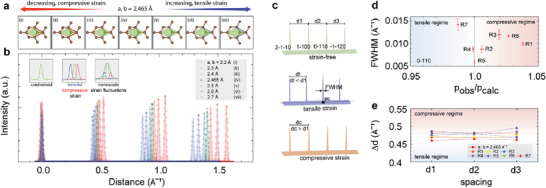
Influence of strain on electron diffraction profile. a) Schematic illustration of changes in the graphene unit cell upon i–iii) decreasing (compressive, red) unit cell length, iv) strain‐free unit cell, and v–vii) increasing (tensile, blue) unit cell length. The changes in the unit cell area due to compressive and tensile strain are highlighted using red and blue regions. b) Representative intensity profiles extracted by simulating the unit cell changes. Inset: Illustration of three conditions depicting strain‐free, presence of only tensile/compressive strain or a combination of both. c) Illustration of diffraction intensity profiles for strain‐free (green circle), tensile (blue circle), and compressive strain (red circle) conditions. Note: *d*_1_, *d*_2_, and *d*_3_ represent the reciprocal spacing between the electron diffraction spots. The equivalent distance *d*_t_ < *d*_1_ decreases (increases, *d*_c_ > *d*_1_) in the presence of tensile (compressive) strain. d) The reciprocal distances *d*_1_, *d*_2_, and *d*_3_ extracted from the intensity profiles of diffractions spots of SAED patterns recorded in regions R1–R7. e) The FWHM versus peak position ratio (p_obs_/p_calc_) of the 0–110 electron diffraction peak for regions R1–R7. The notation pobs denote the peak position of the observed 0–110 diffraction spot, and the p_calc_ denotes the calculated peak position of strain‐free graphene.

In **Figure** **4**a, the changes of compressive (Figure 4ai–iii) and tensile strain (Figure 4av–vii) on the strain‐free graphene unit cell (Figure 4aiv) are presented. The relative decrease in the unit cell due to compressive strain is highlighted in the red region (Figure 4ai–iii). The increase in the unit cell due to tensile strain is highlighted in the blue region (Figure 4av–vii). The intensity profiles of diffraction spot profiles extracted from the simulated SAED patterns under different unit cell conditions are presented in Figure 4b. The extracted intensity profiles are across the diffraction spots 2‐1‐10, 1–100, 0–110, and −1‐120. Note that the 2‐1‐10 diffraction spot is considered as the origin during the analysis. The simulated intensity profile extracted from the SAED pattern of strain‐free graphene is highlighted in green (Figure 4b). The downshift (upshift) of the intensity profiles increases with increasing tensile (compressive) strain (Figure 4b). To quantify the influence of strain, we identified three key parameters of intensity profiles of diffraction spots. These parameters, depicted in Figure 4c, include the spacing between the diffraction spots (d1, d2, and d3), the position of the diffraction peak, and the full‐width‐half‐maximum (FWHM). The extracted FWHM versus the ratio *p*_obs_/*p*_calc_ of the graphene‐on‐grid regions R1–R4 and three additional regions R5–R7 is presented in Figure 4d. The “*p*_obs_” denotes the observed peak position of the TEM spot 0–110, and the “*p*_calc_” denotes the calculated peak position of the strain‐free graphene.

A Gaussian fitting was performed to acquire the peak position and the FWHM. In R2, the extracted FWHM is ≈0.00875 Å^−1^, and the *p*_obs_/*p*_calc_ ratio ≈1, the narrow FWHM and the *p*_obs_/*p*_calc_ ratio ≈1 indicates a strain‐free condition within the imaged area of region R2. In an experiment with exfoliated graphene film, 0–110 diffraction peak with FWHM ≈0.01 Å^−1^ was reported.^[^ ^5^ ^]^ In region R1, a broader FWHM ≈0.01 Å^−1^, and the *p*_obs_/*p*_calc_ ratio ≈1.037 is observable. An increase in the FWHM and the *p*_obs_/*p*_calc_ ratio can be explained by an increase in compressive strain within the imaged region R1. In Figure 3g,i, the simulated intensity profiles of diffractions spot for unit cell, a, b = 2.4 Å overlap with the experimental intensity profiles for region R1 and R3. The *p*_obs_/*p*_calc_ ratio extracted for a graphene‐on‐grid region R1–R6 was ≥ 1, indicating the presence of compressive strain. Conversely, *p*_obs_/*p*_calc_ ratio < 1 suggested the presence of tensile strain within the imaged region of R7 In Figure 4e, the distance between the diffraction spots (D*d*) for all the graphene‐on‐grid regions R1–R7 is presented. The increase in distance (D*d*) between the diffraction spots due to compressive strain is observable in R1–R6 curves (see Figure 4e) compared to the reference (D*d*) extracted from the intensity profile (red square, Figure 4e). A decrease in the D*d* due to tensile strain can be noticed in R7 (red hexagon, Figure 4e). The reference curve refers to the simulated intensity profile with a, b = 2.465 Å. The R2 curve (yellow triangle, Figure 4e) is observable to overlap with the reference curve (red square, Figure 4e).

### 2.3 Intrinsic Compressive Strain and Defect Quantification Using Raman Spectroscopy Across Graphene‐on‐Grid Regions

**Figure 5:**
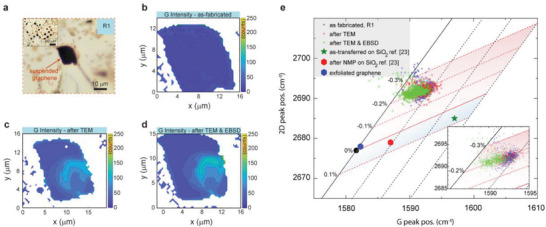
Raman mapping of intrinsic strain within a suspended graphene‐on‐grid region. a) Optical image of the graphene‐on‐grid region R. Inset: Optical image of grid region around region R1 (highlighted using red dotted lines). The G band intensity Raman map of R1 acquired b) as‐fabricated, c) after TEM imaging, and d) after TEM imaging followed by EBSD mapping of the growth substrate. Note: The appearance of partial changes in the R1 G band intensity map after the TEM imaging. e) The 2D peak position (*w*_2D_) versus the G peak position (*w*_G_) of as‐fabricated (blue circle), after TEM (red circle), and after TEM imaging followed by EBSD mapping (green circle). Additional data of as‐transferred CVD graphene on SiO2 (green star), after solvent (NMP) treatment (red hexagon) extracted from ref. [^[^ ^23^ ^]^], and exfoliated graphene on SiO2 (blue hexagon) are presented. Inset: The black line represents the peak position (*w*_2D_, *w*_G_) values for a charge neutral graphene under uniaxial strain (slope ≈2.2). The red line represents the peak position for a doped, strain‐free graphene (slope ≈0.75). The dotted black lines (red line) represent the trajectory of strain (doping density) for a representative doping density (strain).

To validate the presence of the intrinsic compressive strain observed during the TEM characterization, we perform large‐area Raman mapping of graphene‐on‐grid regions. In addition, beam‐induced damage of the graphene‐on‐grid regions was quantified by Raman mapping after graphene‐on‐grid fabrication and before the electron beam exposure. The optical image of the graphene‐on‐grid region R1 is presented in **Figure** **5**a with an inset: the overview of the surrounding grid region. The Raman map of the as‐fabricated graphene G band intensity of region R1 is presented in Figure 5b. It shows a uniform G band intensity across the suspended graphene film. Furthermore, the Raman map of the G band intensity after the TEM imaging of region R1 is presented in Figure 5c. In the bottom right of region R1 (Figure 5c), can be observed an increase in the G band intensity after TEM imaging. The Raman map of the G band intensity of region R1 acquired after the EBSD mapping of the growth substrate is presented in Figure 5d. The electron beam‐induced damage of the graphene film is observable in the Raman map acquired after TEM imaging and EBSD mapping. However, no further increase in the beam‐induced damage area is observable. By performing Lorentzian fit of the D, G, and 2D band, we access the peak intensity, position, and the FWHM of the respective Raman peaks of graphene. The G band intensity maps of additional graphene‐on‐grid regions R1–R7 are presented in Figures S14–S19 (Supporting Information).

The 2D peak position (*w*_2D_) versus the G peak position (*w*_G_) of region R1 after each condition is presented in Figure 5e. The vector model was used to quantify the type and magnitude of strain and doping in single‐layer graphene films.^[^ ^22^ ^]^

This model utilizes the ratio of 2D and G peak positions to establish the primary strain and doping trends. The black circle in Figure 5e represents the strain and doping‐free graphene extracted from an exfoliated, suspended graphene sample with *w*_G_ref1_ ≈1581.6 cm^−1^ and *w*_2D_ref1_ ≈2676.9 cm^−1^ (black circle, Figure 5e).^[^ ^22^ ^]^

The exfoliated sample reference matches closely with our exfoliated graphene sample on SiO_2_ with *w*_G_ref2_ ≈1582.3 cm^−1^ and *w*_2D_ref2_ ≈2677.9 cm^−1^ (blue hexagon, Figure 5e). The solid black line in Figure 5e denotes the 2D and G peak position profile in case of uniaxial strain dominant neutral graphene film, with a 2D/G peak position ratio ≈2.2. The solid red line in Figure 5e indicates the 2D and G peak position profile of a strain‐free graphene film with increasing doping density, with 2D/G peak position ratio of ≈0.75. Each of the black dotted lines indicates an increase in strain along a uniform doping density profile, and each of the red dotted lines indicates an increase in doping density at a fixed strain profile, adapted from Lee et al.^[^ ^22^ ^]^ Note that the full length of the solid red line corresponds to the doping density of ≈0–1.6 × 10^13^ cm^−2^.^[^ ^22^ ^]^ We can observe that the *w*_2D_ and *w*_G_ peak positions of the as‐fabricated sample (blue circle) are along the compressive strain profile ≈−0.2% and a doping density below < 5 × 10^12^ cm^−2^. The 2D/G peak positions of region R1 after TEM imaging (red circle) overlap with the as‐fabricated data. A decrease in doping density is observable with a downshift of the 2D/G peak positions of region R1 after the EBSD mapping. However, the 2D/G peak positions consistently overlap along the compressive strain profile ≈−0.2% and doping profile below < 5 × 10^12^ cm^−2^ across all three conditions. The 2D/G peak position of the suspended graphene‐on‐grid regions was compared with on‐substrate graphene samples to probe the influence of the underlying substrate. For comparison we extract the 2D/G peak position of as‐transferred CVD graphene film on SiO_2_ substrate and after solvent treatment (*n*‐methyl‐2‐pyrrolidone, NMP).^[^ ^23^ ^]^ The average 2D/G peak position with *w*_G_ref3_ ≈1597 cm^−1^ and *w*_2D_ref3_ ≈2685 cm^−1^ (green star, Figure 5e) of an as‐transferred CVD graphene film on SiO_2_ substrate is presented in Figure 5e.^[^ ^23^ ^]^ Note that the 2D/G peak position is in the tensile regime < 0.1% with high doping density > 10^13^ cm^−2^. After solvent exposure, the 2D/G peak position (*w*_G_NMP_ ≈1587 cm^−1^ and *w*_2D_NMP_ ≈2679 cm^−1^, red hexagon, Figure 5e) shifted towards a lower doping density regime < 5 × 10^12^ cm^−2^ but remained within the tensile regime < 0.1%.^[^ ^23^ ^]^

Such observations indicate that the doping density can be reduced in graphene films. However, the substrate‐induced strain could remain persistent. The influence of the sample fabrication process on the doping density is observable. Doping density > 10^13^ cm^−2^ in the as‐transferred graphene film on SiO_2_ (green star, Figure 5e) is ≈2× higher in comparison to as‐fabricated graphene‐on‐grid region R1 (blue circle) < 5 × 10^12^ cm^−2^.

**Figure 6:**
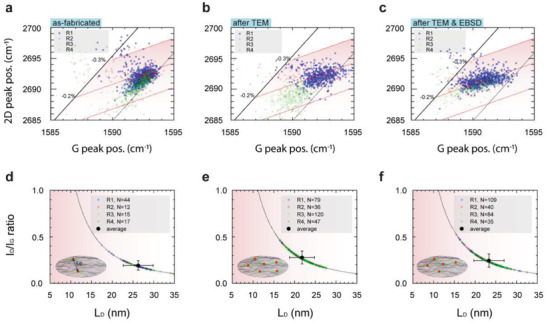
Raman mapping of intrinsic strain and defect density across multiple suspended graphene‐on‐grid regions. The 2D peak position (*w*_2D_) versus the G peak position (*w*_G_) of regions R1 (blue, square), R2 (red, circle), R3 (light green, triangle), and R4 (dark green, inverted triangle) of a) as fabricated, b) after TEM imaging, and c) after TEM imaging and EBSD mapping. The average distance between the defects (*L*_D_) versus the ratio of D to G peak intensity (*I*_D_/*I*_G_) of d) as‐fabricated, e) after TEM, and f) after TEM imaging and EBSD mapping of regions R1–R4. Inset: The black dotted line represents the calculated *L*_D_ versus *I*_D_/*I*_G_ ratio for point defects (*r*_S_ = 1 nm, *r*_A_ = 3 nm, and *l*_L_ = 514 nm). The illustration represents the defect density (red and green regions) and *L*_D_ on the graphene film at each processing condition.

The 2D and G peak position of multiple graphene‐on‐grid regions R1–R4 was compared in **Figure** **6**a–c. In Figure 6a, the 2D and G peak positions of as‐fabricated graphene‐on‐grid regions R1–R4 are presented. Similar to the observation in Figure 5e, the presence of compressive strain in the range of −0.1–−0.2% and a doping density of <5 × 10^12^ cm^−2^ is observable in the as‐fabricated graphene‐on‐grid regions R2 (red circle), R3 (green triangle), and R4 (green inverted triangle). However, the 2D and G peak positions of regions R3 and R4 are aligned along the black dotted line, indicating the presence of a broader intrinsic strain in the range within ≈−0.1–−0.2% and a narrow doping density. The 2D and G peak positions collected after TEM imaging of graphene‐on‐grid regions R1–R4 are presented in Figure 6b. The 2D and G peak positions of R3 and R4 are aligned closer to the red dotted line, i.e., along the −0.2% compressive strain axis after exposure to the electron beam during TEM imaging. The profile of the 2D to G peak positions of regions R1–R4 indicates a broader doping density and intrinsic strain in the range of ≈−0.1–−0.2%. In Figure 6c, the 2D and G peak positions collected after the EBSD mapping of the growth substrate is presented. We can observe a uniform alignment of the 2D & G peak position along the −0.2% compressive strain axis with broader doping density profile within the graphene‐on‐grid regions. The broader doping density profile indicates the presence of local graphene regions with lower doping density compared to the as‐fabricated sample (Figure 6a). The G and 2D peak positions of all the graphene‐on‐grid regions R1–R7 after each processing condition are presented in Figures S20–S22 (Supporting Information).

Collecting the Raman spectra allows for quantifying the defect density profile in the graphene‐on‐grid regions. Defects can be characterized as intrinsic (point‐like defects) and extrinsic defects (induced by processing) in the graphene film.^[^ ^24^, ^25^ ^]^

Length scale such as *L*_D_, denoted as the average distance between point‐like defects, can be related to the D to G band intensity ratio. Considering long‐range disorder and point‐like defects with disorder area (*r*_S_ = 1 nm), and disorder radius around the disorder *r*_A_ = 3 nm, the following equation is used to quantify the average defect length:^[^ ^24^ ^]^

(1) $$L_{D}^{2}\;\left(nm^{2}\right)=\left(1.8\;\pm \;0.5\right)\;\times 10^{-9}\lambda_{L}^{4}{\left(\frac{I_{D}}{I_{G}}\right)}^{-1}$$

The term *λ*_L_ denotes the laser wavelength (514 nm), and the *I*_D_/*I*_G_ is the intensity ratio of the D, and G, Raman peaks. Using Equation 1, we can estimate the *I*_D_/*I*_G_ versus the average defect length *L*_D_ profile, highlighted as the black dotted line in Figure 6d–f. In Figure 6d, the *I*_D_/*I*_G_ versus the *L*_D_ is presented for the as‐fabricated graphene‐on‐grid regions R1–R4. The average *I*_D_/*I*_G_ ratio across graphene‐on‐grid regions R1–R4 is ≈0.19 ± 0.05 in the as‐fabricated graphene‐on‐grid regions, indicating an average defect length L_D_ ≈26 ± 3.6 nm (black hexagon, Figure 6d). After TEM imaging the average *I*_D_/*I*_G_ ratio increased to ≈0.27 ± 0.07. The average defect length decreased to L_D_ ≈21.8 ± 3 nm (Figure 6e). The increase in the *I*_D_/*I*_G_ ratio and shorter defect length *L*_D_ indicates electron beam‐induced damage during the TEM imaging. The average *I*_D_/*I*_G_ ratio after TEM and EBSD imaging is ≈0.24 ± 0.07, with an average defect length *L*_D_ ≈23 ± 3.6 nm (black hexagon, Figure 6f). The increase in defects is further observable with the increase in quantity “N” in each sample type. In the as‐fabricated graphene regions R1–R4, up to 88 *I*_D_/*I*_G_ ratios were estimated. After TEM and EBSD analysis of graphene‐on‐grid regions, up to 282 and 268 *I*_D_/*I*_G_ ratios were estimated. Note the ≈3× increase in defects observable after exposure to the electron beam during TEM analysis.

## 3 Discussion

We have demonstrated an approach to fabricate high‐density suspended graphene‐on‐grid directly on their growth substrate. This approach provides the possibility to study the intrinsic strain and doping in graphene films on their growth substrate and without being influenced by any transfer process. Note that copper orientation‐dependent interaction between the graphene layer and its underlying growth substrate has been reported using Raman spectroscopy.^[^ ^16^ ^]^ By suspending the graphene films, it is possible to minimize such interactions between the graphene layer and the growth substrate. The presence of the growth substrate around the suspended graphene films provides the possibility to correlate the copper substrate orientation with the rotation of the suspended graphene grain. Frank et. al., reported on the interaction of the CVD graphene films with different copper grain orientation. They reported on the dependence of FWHM of 2D peak and the underlying Cu grain orientation.^[^ ^26^ ^]^ In our study, a misorientation angle of up to ≈60° is observable when transitioning from R1 to R2 or R3 to R4 copper grains (see Figures S12 and S13, Supporting Information). Such variation in crystal orientations of the copper grains influence the growth kinetics of graphene and, subsequently, the rotation of graphene grain.^[^ ^20^, ^21^ ^]^ This is evident in rotation angle differences of the SAED patterns presented in Figure 3a–f. However, the misorientation angles within individual copper grains are typically < 1°, indicating homogeneous copper grains with low defect density (see Figure S11, Supporting Information). It could not be analyzed further whether the graphene orientation is consistently inherited from the copper crystal orientation, because 1) the etched openings in the copper substrate typically developed at a copper grain boundary, and 2) the copper grains are much larger in extend than the etched openings with suspended graphene. The presence of six‐fold symmetry in the SAED patterns and the FFT of the TEM image indicates the cleanliness of the graphene‐on‐grid regions. Given the sensitivity of the SAED technique to material properties, we can probe the intrinsic strain in these suspended graphene regions. A consistent compressive strain is observable in the graphene‐on‐grid regions.

However, in the case of region R2 and R4, strain‐free graphene unit cell parameters a, b = 2.465 Å can be overlapped with the collected intensity profiles. The influence of compressive strain in graphene film on SAED pattern is quantified by characterizing the changes in the peak position (*p*_obs_) and the FWHM of the diffraction spot 0–110. A narrow FWHM of ≈0.00875 and ≈0.006 Å^−1^ is observable in regions R2 and R5, for strain‐free graphene region.^[^ ^5^ ^]^ Increasing compressive strain results in an increase of the distance between the diffraction spots and in the peak position (i.e., *p*_obs_/*p*_calc_ > 1 regime). This is observable in Figure 4d, with a gradual increase in FWHM and the ratio of (*p*_obs_/*p*_calc_) in regions R3 (0.012 Å^−1^) and R1 (0.01 Å^−1^). However, such observations are possible in the case of high signal‐to‐noise (SNR) ratio of the SAED patterns. The presence of surface adsorbents minimizes the SAED SNR significantly and limits the observation of the peak position versus FWHM trends observable in cleaner graphene‐on‐grid regions with high SNR SAED patterns. Out‐of‐plane deformation of the graphene film can lead to broadening of the FWHM of the diffraction spot.^[^ ^5^ ^]^ In case of tensile strain, a decrease in the peak position (*p*_obs_/*p*_calc_ < 1 regime) is expected. The presence of both out‐of‐plane deformation and strained graphene regions is likely in our samples. The presence of compressive strain in graphene‐on‐grid regions R1–R4 is further verified using large‐area Raman mapping. The 2D to G peak position indicates the presence of a compressive strain in the range of ≈−0.1– −0.2% and the presence of a doping density below < 5 × 10^12^ cm^−2^. Although the approach presented in Figure 5e is found to be in agreement with uniaxial strain conditions, the presence of biaxial strain in the samples is not excluded.^[^ ^22^ ^]^ The consistent compressive strain can possibly originate from the thermal expansion coefficient mismatch of graphene on copper substrate.^[^ ^27^ ^]^ The presence of doping in our samples is attributed to the sample preparation method, which involves exposure to the developer solution during the UV lithography, and the copper etchant. Such processing conditions are known to induce a p‐type doping in graphene films and have been studied extensively and can be overcome using electrical characterization.^[^ ^8^, ^23^, ^28^ ^]^ Considering similar processing methods followed in our case, we attribute the doping density primarily to hole doping.^[^ ^23^ ^]^ In addition, the limitations of the etching process must be carefully considered during the graphene characterization.

By performing the large‐area Raman mapping, we can study the influence of electron beam exposure on graphene films locally. A defect‐induced Raman peak (D peak) increase is consistently observed in graphene‐on‐grid regions R1–R4. By considering the D to G peak intensity ratio (*I*_D_/*I*_G_), the average defect length (*L*_D_) is estimated. About three times increase in the defect density (*N*) and a decrease in the average defect length (*L*_D_) is observed after the TEM imaging. Raman mapping of regions R1–R4 performed after the EBSD mapping of the growth substrate indicates the defect density (*N*) and *L*_D_ remain higher than the as‐fabricated sample. This is indicative of possible electron beam‐induced damage of the graphene region locally.^[^ ^25^ ^]^

However, the doping density profile of the graphene regions changes considerably between as fabricated and after characterization using TEM and EBSD. The doping density profile is narrow in the as‐fabricated regions R1–R4 with a broader strain range ≈−0.1%–−0.2%. However, after TEM imaging and EBSD mapping, the doping density profile is broader, indicating the presence of graphene regions with lower hole doping density ≈2 × 10^12^ cm^−2^ and aligned along the compressive strain axis of ≈−0.2%. Such a decrease in hole doping density is attributed to the thermal‐induced desorption of surface contamination under low vacuum conditions during the electron beam exposures. Under imaging conditions with acceleration voltages of 80 kV, the transformation of amorphous carbon material into carbon onions on graphene has been reported.^[^ ^29^ ^]^ Lin et al.^[^ ^30^ ^]^ have reported that the introduction of amorphous carbon material on graphene layer can occur during CVD synthesis. This finding helps to explain the increase in the defect density (*N*) and the decrease in average defect length *L*_D_ observed after TEM imaging. Further characterization using low‐energy electron diffraction (LEED) can be beneficial to quantify CVD graphene quality and complement the current findings.^[^ ^31^, ^32^ ^]^ Although, doping and strain properties of our graphene films can be characterized using the methods presented in our work, extensive atomic‐scale characterization is essential and complementary to assess the graphene quality.

In conclusion, by combining transmission electron microscopy and Raman spectroscopy methods, we quantify the intrinsic strain and doping properties and assess the quality of the graphene films. Compressively strained graphene regions are identifiable by their salient features in the SAED patterns. A method to directly identify the type and magnitude of strain is determined by comparing the FWHM, the peak position, and the spacing between the electron diffraction spots. Using large‐area Raman mapping, we confirm the presence of compressive strain and the local influence of electron beam exposure on graphene films. Our approach provides extensive insight into the local, intrinsic strain, and doping profile of the graphene films and is extendable to study other 2D materials directly on their growth substrates. These customized graphene‐on‐grids can also be directly used as ultra‐thin substrates with high SNR for atomic‐resolution imaging and liquid cell TEM. The polymer‐free approach provides cleaner, suspended single‐layer graphene films beneficial for studying graphene's intrinsic thermal, optical, and mechanical properties without the influence of transfer methods.

## 4 Experimental Section

### CVD Graphene Synthesis

A low‐pressure, hot wall CVD reactor from Graphene Square Inc. was used for the CVD graphene synthesis on thin copper foils (≈25 µm thickness). CVD synthesis of graphene was performed at 1000 °C using a methane/hydrogen ratio of ≈1:13 (growth time ≈45 min). After the CVD process, the chamber was cooled to room temperature in argon and hydrogen gas flow. The graphene film on the bottom side of the copper foil was removed using an oxygen plasma exposure (30 W, 45 s, 30/15 sccm of Oxygen/Argon).

### Graphene‐on‐Grid Fabrication

A three‐step process was followed to prepare the high‐density CVD graphene‐on‐grid array on copper (CVDG/Cu). In the first step, a UV PL process was used to fabricate a high‐density graphene‐on‐grid array on the bottom side of the copper growth substrate (CVDG/Cu/polymer). A layer of AZ photoresist layer was spin‐coated on the bottom side of the CVDG/copper substrate (4000 rpm, 40 s). During the second step, the polymer mask allows for selective etching of the copper growth substrate. The CVDG/copper/AZ substrate was placed with AZ layer in contact with ammonium persulphate solution (APS, 50 mm) to etch the copper grid regions. After the etching of the copper regions, the CVDG/Cu/AZ layer was transferred to DIW solution to remove etchant residues and then transferred to IPA. The AZ layer was removed in acetone and transferred to IPA. A critical point drying process (Tousimis CPD 931) was performed to suspend the single‐layer CVD graphene, and dry the suspended graphene‐on‐grid, and finally punched to 3 mm diameter (see: Figure S1, Supporting Information).

### Raman Characterization

The graphene‐on‐grid regions were characterized using an inVia Renishaw microscope using 514 nm excitation wavelength. The Raman mapping was performed using an objective of ≈100× (NA = 0.85), and each spectrum was collected using a laser power ≈0.5 mW, exposure time ≈0.1 s, and ten accumulations. The Raman peak features were analyzed using Matlab.

### TEM Characterization

All the selected area electron diffraction (SAED), (scanning) transmission electron microscopy ((S)/TEM), and the electron energy loss spectroscopy (EELS) measurements were performed using JEOL JEM CFEG F200 operated at 80 kV acceleration voltage.

### Intensity Profile Extraction and Simulation

Gatan Digital Micrograph software was used to extract the intensity profiles of diffraction spots along the specific direction indicated on images of SAED patterns, which were acquired on the graphene‐on‐grid regions, R1–R7. The diffraction peaks parameters were analyzed using OriginLab.

The reference intensity profile of diffraction spots along specific direction for single‐layer graphene, was extracted by constructing a graphene layer with space group: P 6_3_ m c, lattice parameters a, b = 2.465 Å, acceleration voltage: 80 kV, and camera length: 100 cm using SingleCrystal software.

### EBSD Characterization

The crystal orientation maps of the copper substrate were collected using an SEM Quanta 200F with EDAX Hikari EBSD detector with 30 kV acceleration voltage. Scanning arrays of 300 by 250 µm maps with a step size of 1 µm.

## Conflict of Interest

The authors declare no conflict of interest.

## Supporting information

Supporting Information

## Data Availability

The data that support the findings of this study are available from the corresponding author upon reasonable request.
