# Supplementary material for: Probing the Intrinsic Strain in Suspended Graphene Films Using Electron and Optical Microscopy
Source: Adv Sci (Weinh). 2023 Dec 6;11(5):2305366. doi: 10.1002/advs.202305366 (PMC10837373; doi:10.1002/advs.202305366)
Supplement: Supplementary file 1 — Supporting Information [file ADVS-11-2305366-s001.pdf]

ADVANCED SCIENCE
Open Access

Supporting Information

for *Adv. Sci.*, DOI 10.1002/advs.202305366

Probing the Intrinsic Strain in Suspended Graphene Films Using Electron and Optical Microscopy

Kishan Thodkar, Milivoj Plodinec, Fabian Gramm and Karsten Kunze*

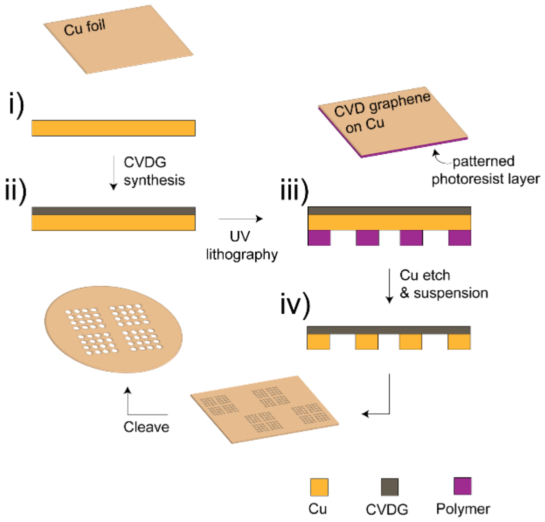

Figure S1. Schematic of the high-density graphene-on-grid fabrication process. **i-ii)** CVD graphene synthesis is performed on copper foils. **iii)** High-density etch windows are realized on the bottom side of the copper foil using a photolithography process. **iv)** The exposed copper regions are etched, and the graphene grids are suspended using critical point drying process. Individual grids are then punched into ~3 mm diameter grids.

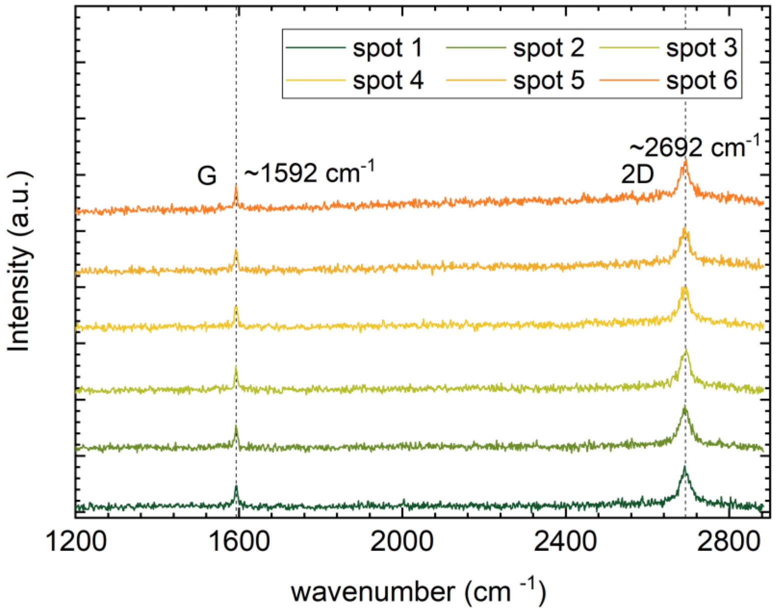

Figure S2. Representative Raman spectra collected within graphene-on-grid region R5.

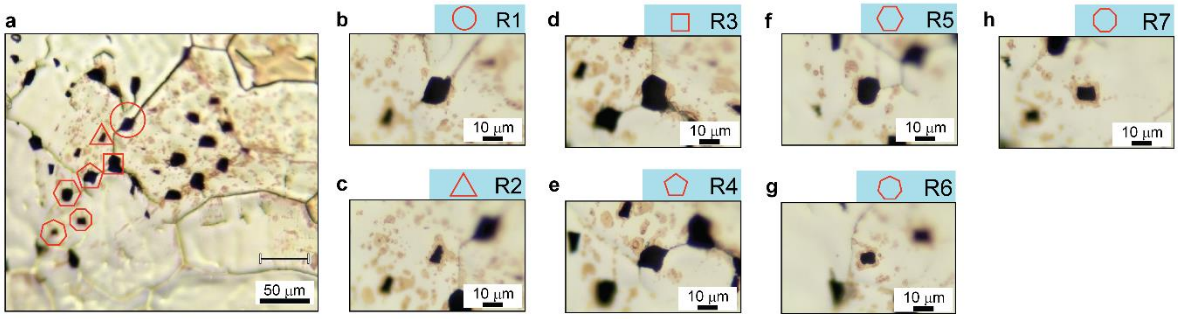

Figure S3. Optical images of **(a)** overview of a region with freely suspended graphene on the grid (marked by red shapes), **(b)** R1 (circle), **(c)** R2 (triangle), **(d)** R3 (square), **(e)** R4 (pentagon), **(f)** R5 (hexagon), **(g)** R6 (heptagon), and **(h)** R7 (octagon).

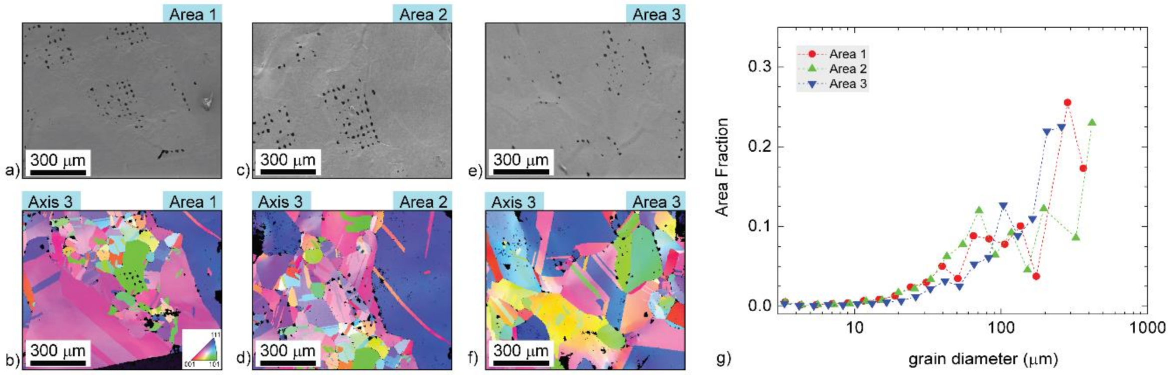

Figure S4. SEM and EBSD characterization of the copper growth substrate used for the suspended graphene-on-grid fabrication. **(a, c, e)** SEM secondary electron images of area 1-3, and **(b, d, f)** crystal orientation maps of area 1-3 (inset in b) indicates the color legend for crystal directions aligned normal to the foil surface). **(g)** Grain size distribution by area fraction versus grain diameter of the copper growth substrate of areas 1-3.

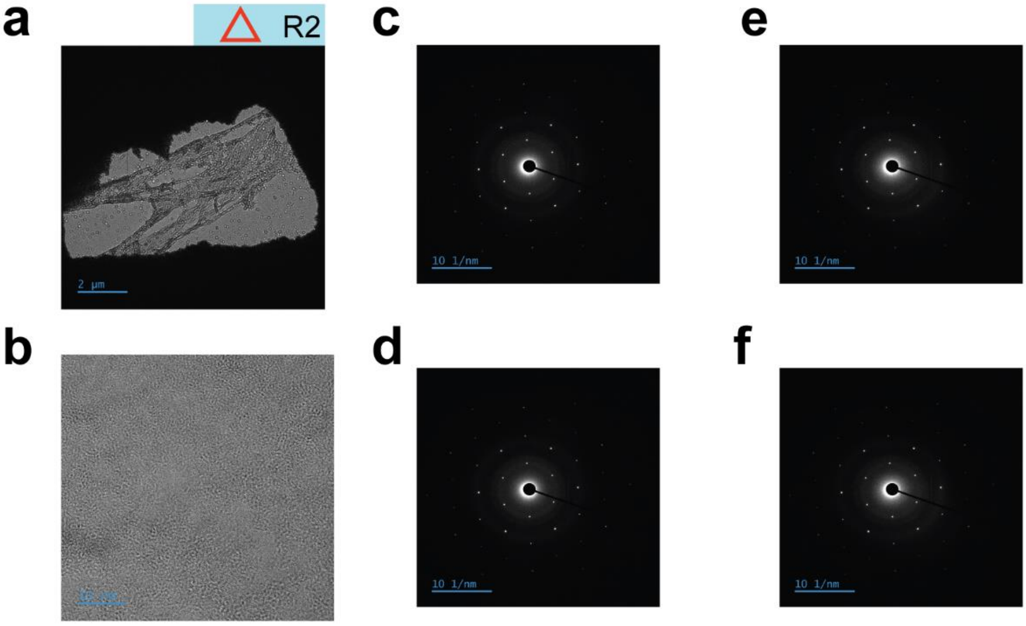

Figure S5. SAED patterns of graphene grid region R2. TEM images of graphene grid region R2 recorded at low **(a)** and high **(b)** magnification. **(c-f)** The SAED patterns collected within the graphene grid region R2.

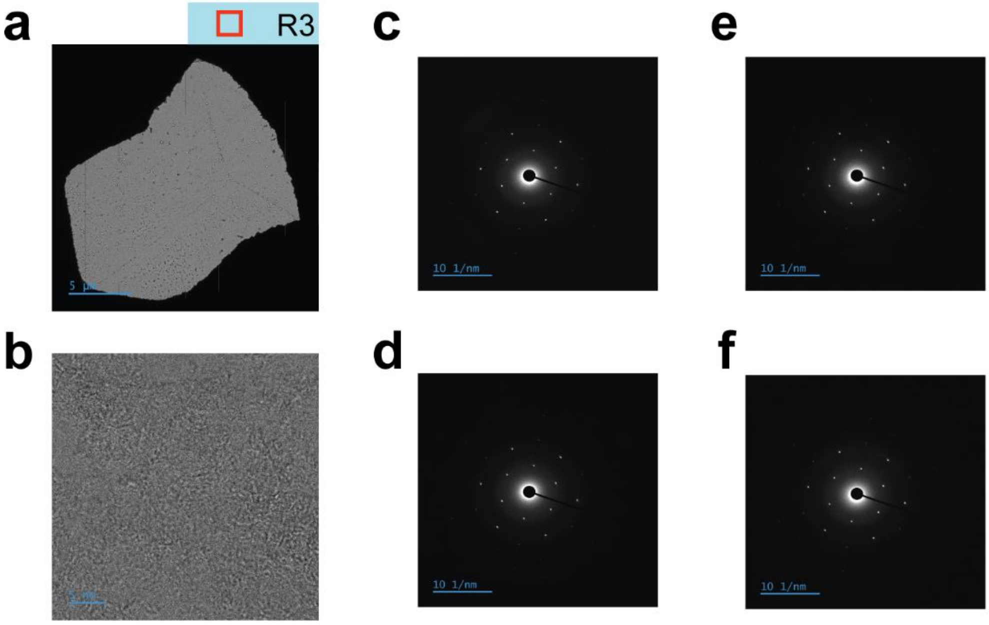

Figure S6. SAED patterns of graphene grid region R3. TEM images of graphene grid region R3 recorded at low **(a)** and high **(b)** magnification. **(c-f)** The SAED patterns collected within the graphene grid region R3.

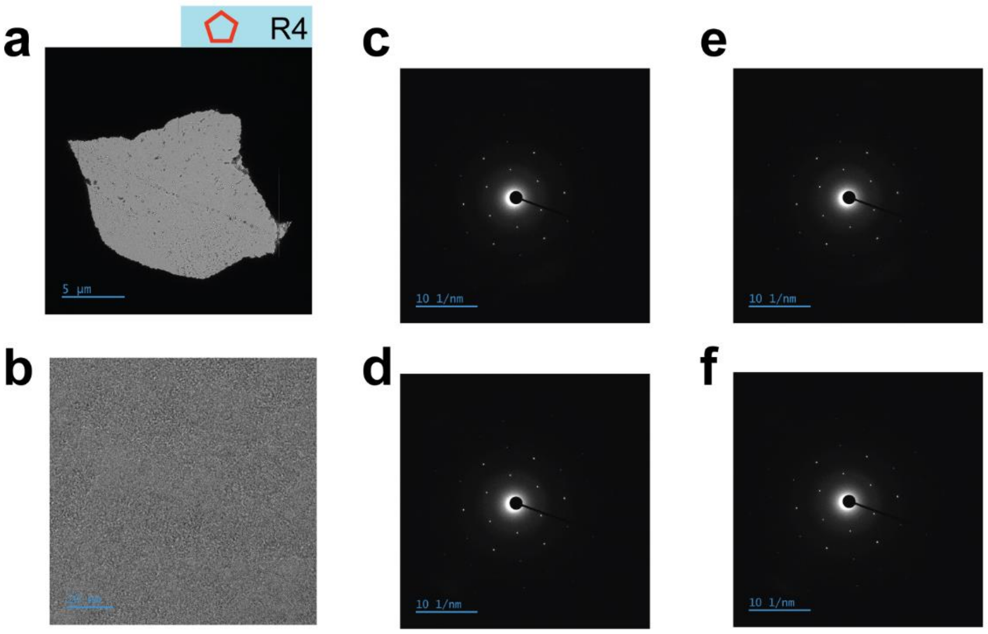

Figure S7. SAED patterns of graphene grid region R4. TEM images of graphene grid region R4 recorded at low **(a)** and high **(b)** magnification. **(c-f)** The SAED patterns collected within the graphene grid region R4.

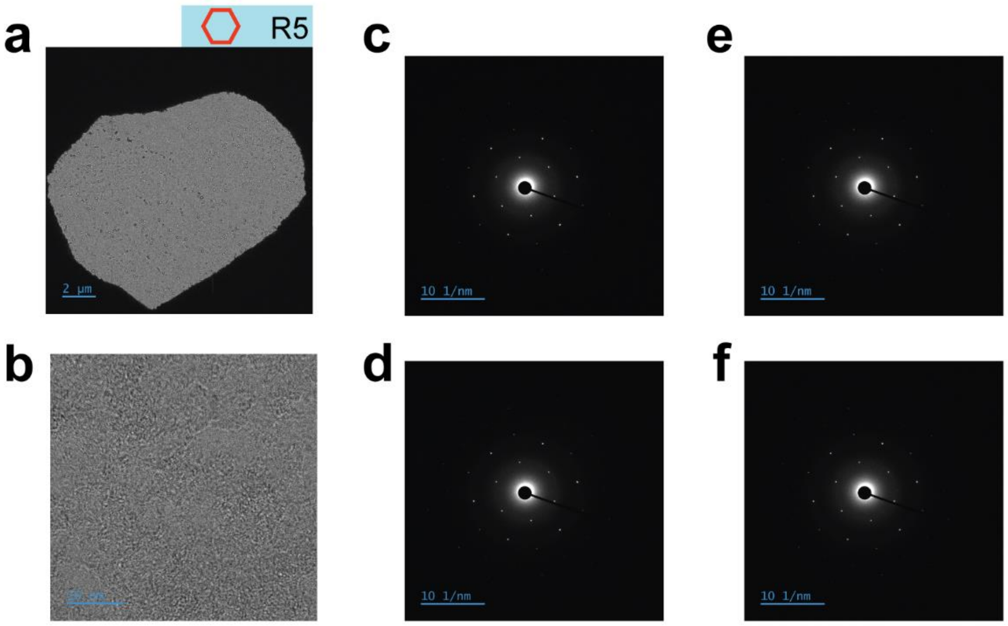

Figure S8. SAED patterns of graphene grid region R5. TEM images of graphene grid region R5 recorded at low **(a)** and high **(b)** magnification. **(c-f)** The SAED patterns collected within the graphene grid region R5.

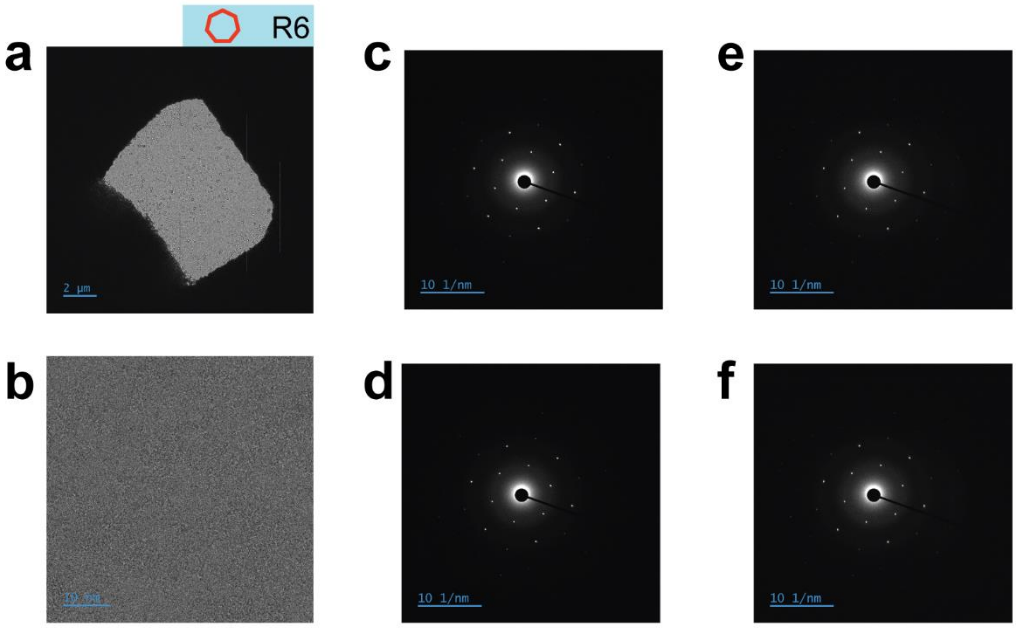

Figure S9. SAED patterns of graphene grid region R6. TEM images of graphene grid region R6 recorded at low **(a)** and high **(b)** magnification. **(c-f)** The SAED patterns collected within the graphene grid region R6.

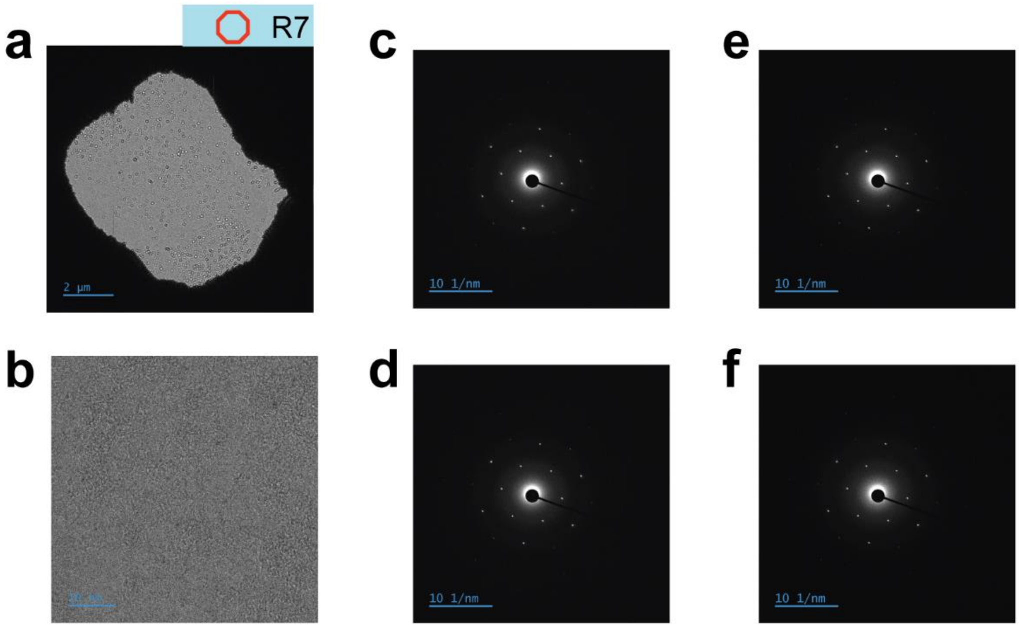

Figure S10. SAED patterns of graphene grid region R7. TEM images of graphene grid region R7 recorded at low **(a)** and high **(b)** magnification. **(c-f)** The SAED patterns collected within the graphene grid region R7.

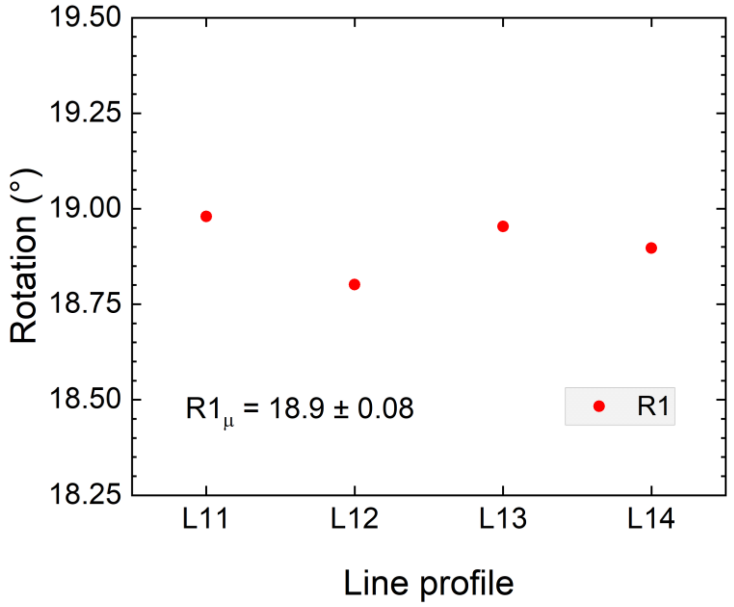

Figure S11. The rotation angles (L11-L14) collected from the SAED patterns acquired within an individual graphene grid region R1 presented in figure 2.

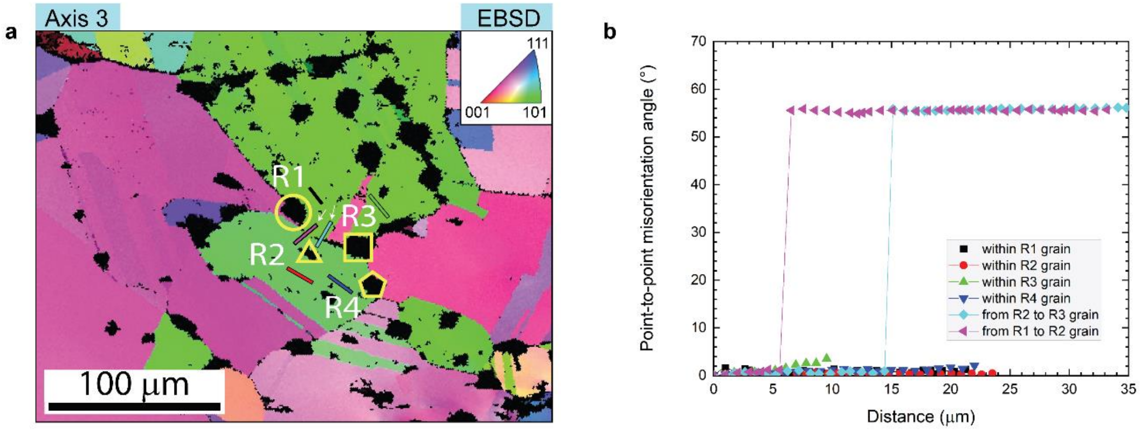

Figure S12. **(a)** Crystal orientation map of the copper growth substrate around the graphene grid region (area ~300x250 μm^2) considered during this work (inset indicates the color legend for crystal directions aligned normal to the foil surface). Inset indicates the color legend for crystal directions aligned in-plane to the foil surface, along axis 3. **(b)** The point-to-point misorientation angle (degrees) along various profiles within single copper grains (black, red, green, and blue lines) and when transitioning from different grains (cyan between R2 and R3, magenta between R1 and R2).

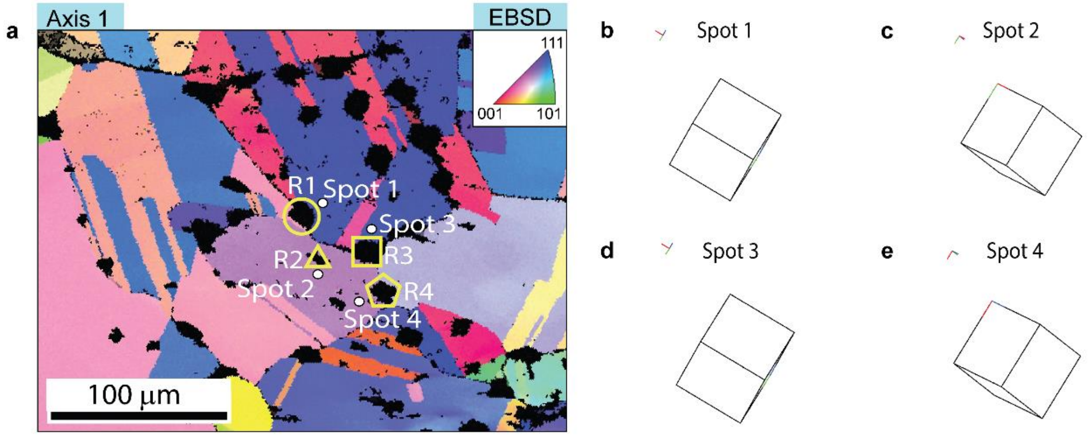

Figure S13. **(a)** Crystal orientation map of the copper growth substrate around the graphene grid region R1-R4 considered during this work. Inset indicates the color legend for crystal directions aligned in-plane to the foil surface, along axis 1. **(b-e)** Wireframe sketches of the copper grain orientation at spot 1-4 next to the graphene grid regions R1-R4.

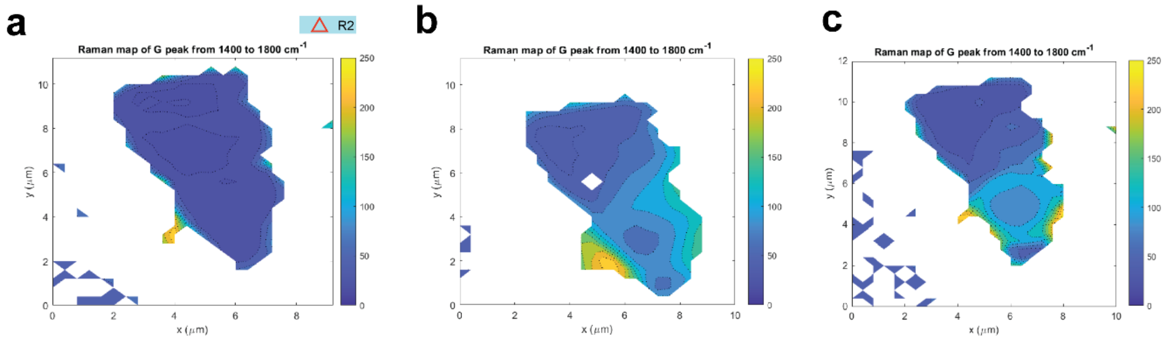

Figure S14. The G band intensity Raman maps of the graphene grid regions R2 **(a)** as fabricated, **(b)** after TEM imaging, and **(c)** after TEM and EBSD imaging.

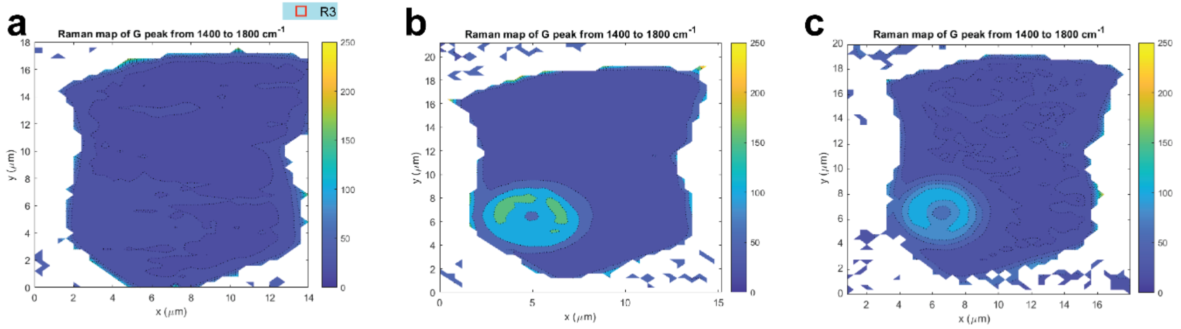

Figure S15. The G band intensity Raman maps of the graphene grid regions R3 **(a)** as fabricated, **(b)** after TEM imaging, and **(c)** after TEM and EBSD imaging.

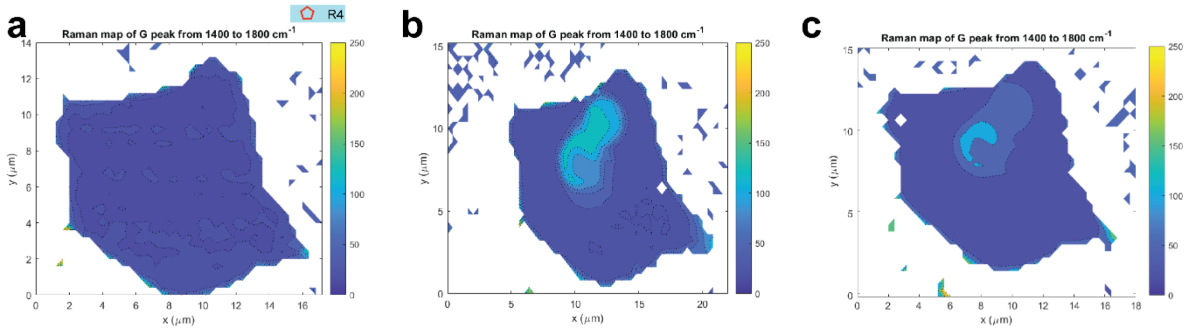

Figure S16. The G band intensity Raman maps of the graphene grid regions R4 **(a)** as fabricated, **(b)** after TEM imaging, and **(c)** after TEM and EBSD imaging.

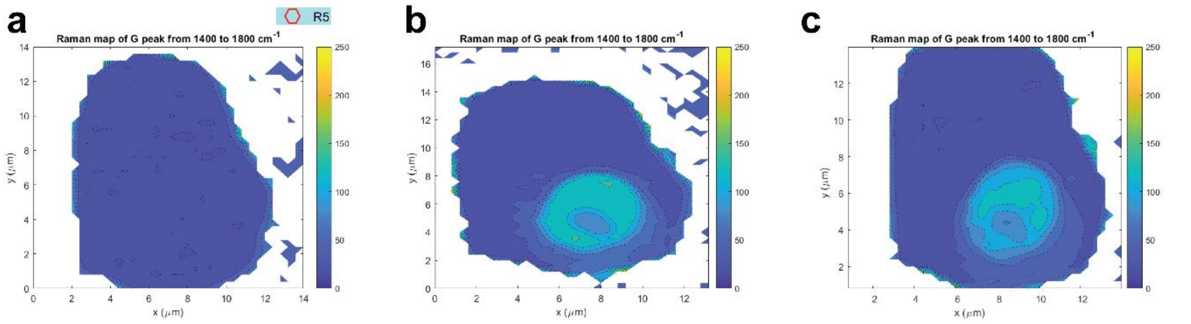

Figure S17. The G band intensity Raman maps of the graphene grid regions R5 **(a)** as fabricated, **(b)** after TEM imaging, and **(c)** after TEM and EBSD imaging.

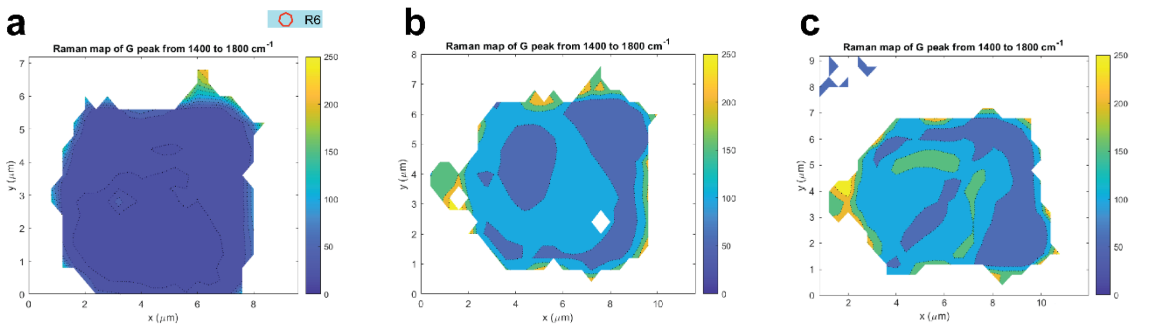

Figure S18. The G band intensity Raman maps of the graphene grid regions R6 **(a)** as fabricated, **(b)** after TEM imaging, and **(c)** after TEM and EBSD imaging.

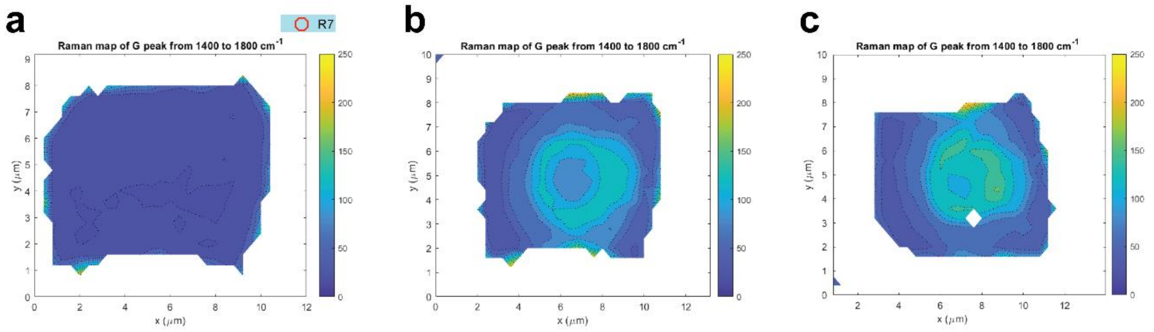

Figure S19. The G band intensity Raman maps of the graphene grid regions R7 **(a)** as fabricated, **(b)** after TEM imaging, and **(c)** after TEM and EBSD imaging.

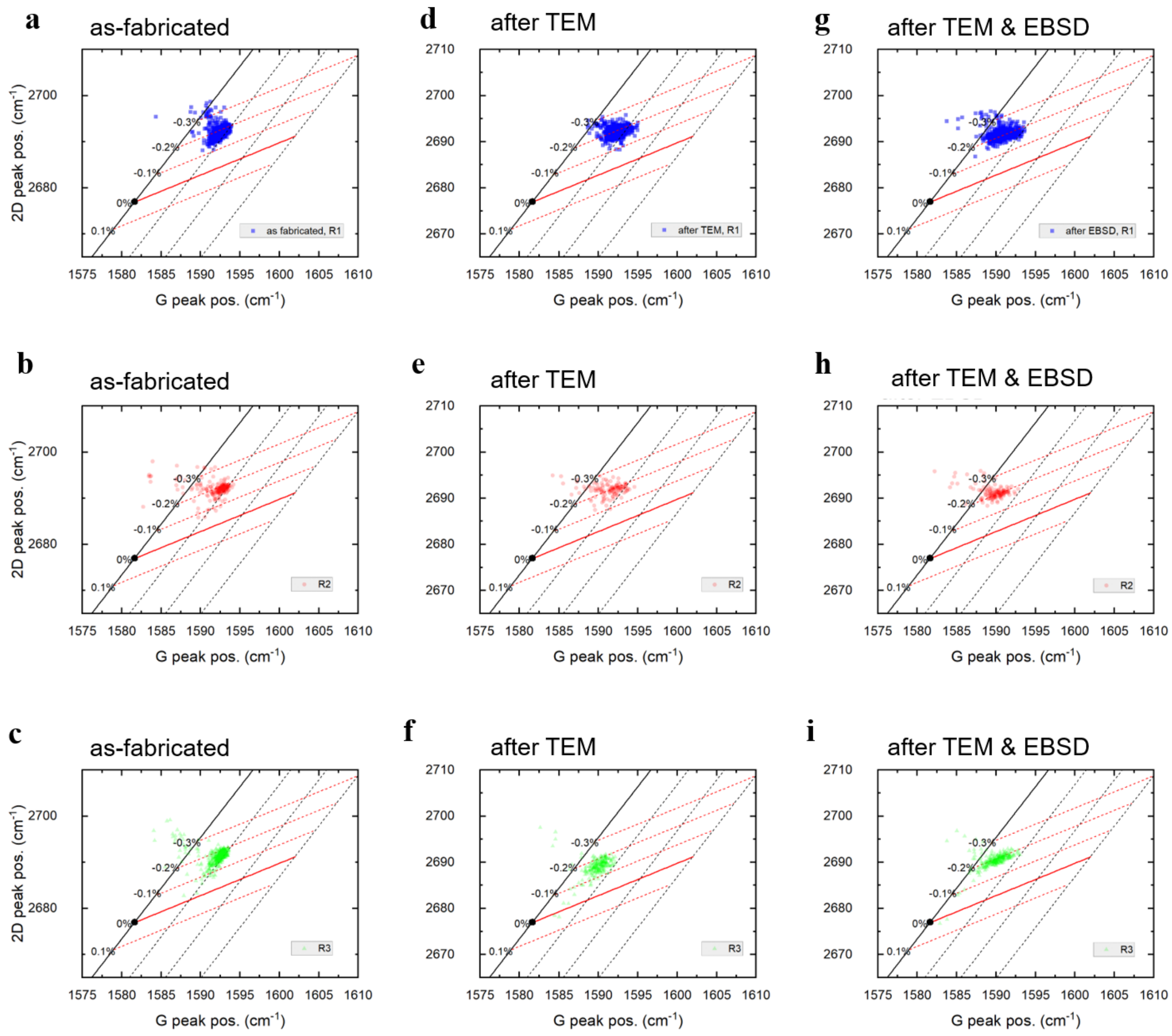

Figure S20. The G versus 2D peak positions of **(a-c)** as fabricated, **(d-f)** after TEM imaging, and **(g-i)** after TEM and EBSD imaging of the graphene grid regions R1-R3.

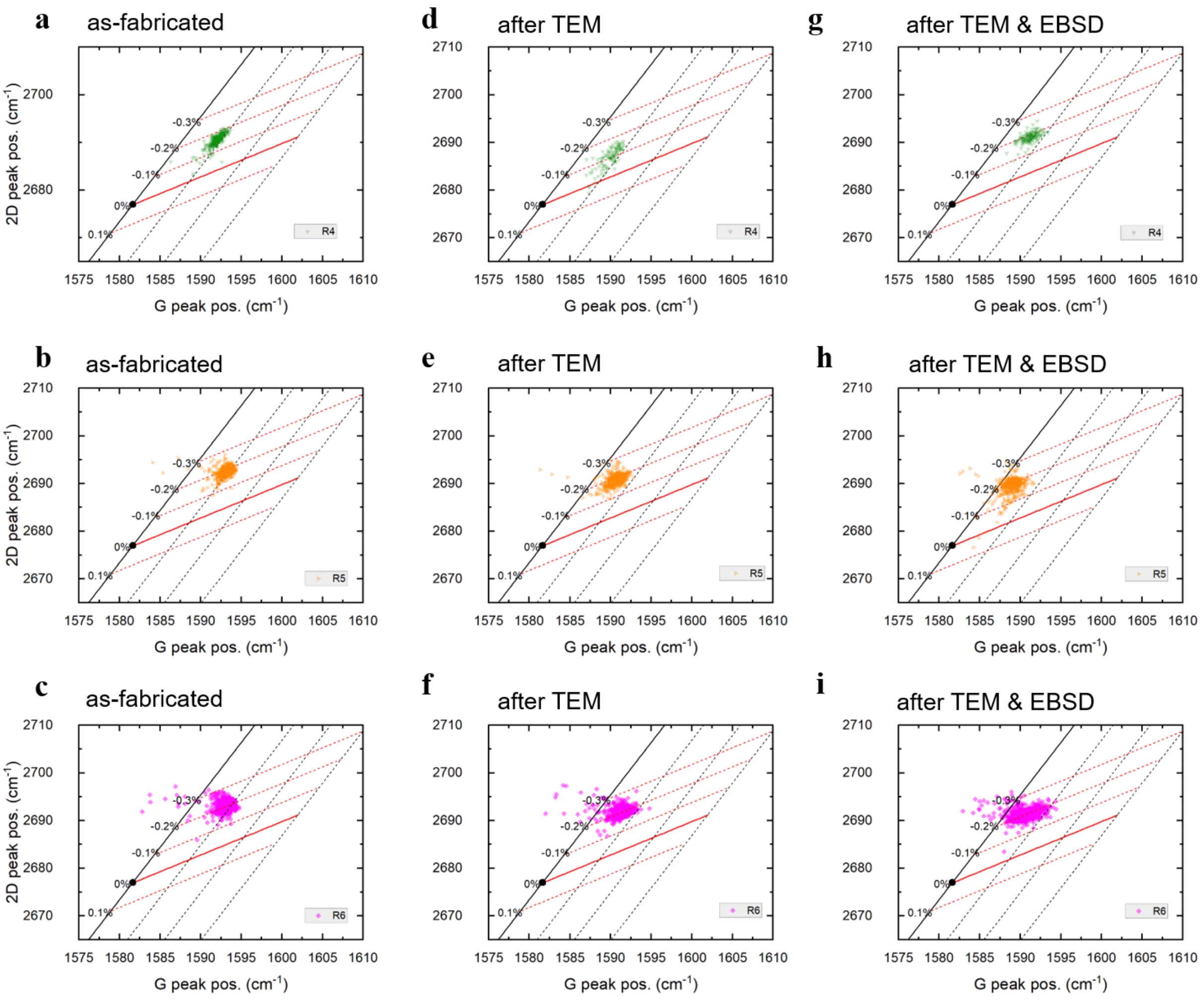

Figure S21. The G versus 2D peak positions of **(a-c)** as fabricated, **(d-f)** after TEM imaging, and **(g-i)** after TEM and EBSD imaging of the graphene grid regions R4-R6.

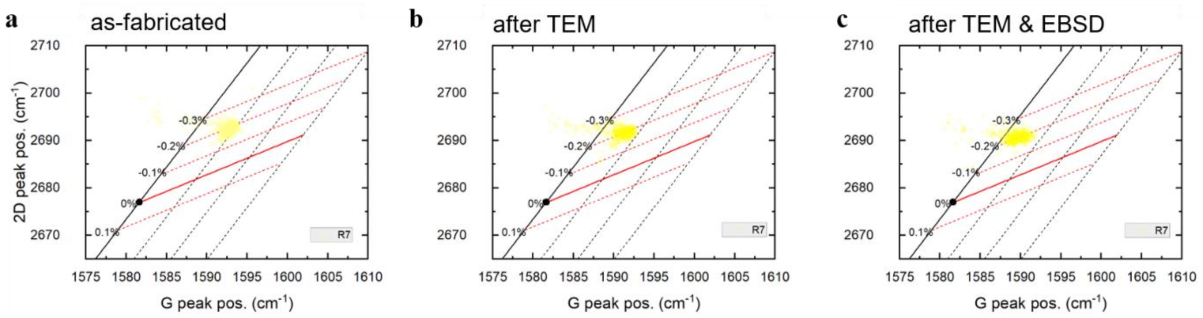

Figure S22. The G versus 2D peak positions of **(a)** as fabricated, **(b)** after TEM imaging, and **(c)** after TEM and EBSD imaging of the graphene grid regions R7.
